# THE POTENTIAL FOR MIND–BODY THERAPIES AS FRAILTY INTERVENTIONS

**DOI:** 10.1093/geroni/igae098.1279

**Published:** 2024-12-31

**Authors:** Julia Loewenthal

**Affiliations:** Brigham and Women’s Hospital, Boston, Massachusetts, United States

## Abstract

Strategies to prevent and manage frailty are priorities in clinical medicine and public health. Current evidence-based interventions for frailty are multimodal in nature, with physical activity as a key element. Mind-body therapies, especially those that incorporate movement such as yoga and tai chi, include physical postures, breathing practices, meditation, and other elements. In our recent systematic review of 33 randomized controlled trials of yoga interventions in older adults (2384 participants), there was moderate-certainty evidence that yoga improved gait speed and lower extremity strength when compared to inactive control groups. Both yoga and tai chi are known to improve physical function, even in already frail populations. Emerging evidence suggests they may modulate cellular and molecular hallmarks of aging, especially inflammation. Mind-body therapies have additional benefits for cognition, mood, and sleep. They likely confer resilience by positively impacting core systems for maintaining homeostasis, including musculoskeletal, metabolic, and stress-response systems. They are not only highly adaptable for older populations, but also increasingly popular: among US adults, use of yoga alone increased from 5% in 2012 to 15.8% in 2022. They may be delivered in a group format to reduce social isolation, and are increasingly accessible via telehealth, senior centers, and other community-based organizations. In addition, there is evidence that mind-body therapies are an entry point to other types of physical activity, especially for women. They are promising frailty interventions, though more work is needed to understand optimal dose, duration, and effectiveness.

